# Patient Experienced Continuity of Care in the Psychiatric Healthcare System—A Study Including Immigrants, Refugees and Ethnic Danes

**DOI:** 10.3390/ijerph110909739

**Published:** 2014-09-17

**Authors:** Natasja Koitzsch Jensen, Katrine Schepelern Johansen, Marianne Kastrup, Allan Krasnik, Marie Norredam

**Affiliations:** 1 The Danish Research Centre for Migration, Ethnicity and Health (MESU), Section for Health Services Research, Department of Public Health, Copenhagen University, Øster Farimagsgade 5, Copenhagen 1014, Denmark; E-Mails: alk@sund.ku.dk (A.K.); mano@sund.ku.dk (M.N.); 2 KORA, The Danish Institute for Local and Regional Government Research, Købmagergade 22, Copenhagen 1150, Denmark; E-Mail: kajo@kora.dk; 3 Competence Center for Transcultural Psychiatry, Psychiatric Center Ballerup, Niels Andersens Vej 65, Hellerup 2900, Denmark; E-Mail: marianne.kastrup@dadlnet.dk; 4 Section of Immigrant Medicine, Department of Infectious Disease, Copenhagen University Hospital, Hvidovre Hospital, Kettegård Alle 30, Hvidovre 2650, Denmark

**Keywords:** psychiatry, mental illness, migration, immigrants, refugees, health care system, continuity of care, illness narratives

## Abstract

*Aim:* The purpose of this study was to investigate continuity of care in the psychiatric healthcare system from the perspective of patients, including vulnerable groups such as immigrants and refugees. *Method*: The study is based on 19 narrative interviews conducted with 15 patients with diverse migration backgrounds (immigrants, descendents, refugees, and ethnic Danes). Patients were recruited from a community psychiatric centre situated in an area with a high proportion of immigrants and refugees. Data were analysed through the lens of a theoretical framework of continuity of care in psychiatry, developed in 2004 by Joyce *et al.*, which encompasses four domains: accessibility, individualised care, relationship base and service delivery. *Results*: Investigating continuity of care, we found issues of specific concern to immigrants and refugees, but also commonalities across the groups. For accessibility, areas pertinent to immigrants and refugees include lack of knowledge concerning mental illness and obligations towards children. In terms of individualised care, trauma, additional vulnerability, and taboo concerning mental illness were of specific concern. In the domain of service delivery, social services included assistance with immigration papers for immigrants and refugees. In the relationship base domain, no differences were identified. *Implications for priority area*: The treatment courses of patients in the psychiatric field are complex and diverse and the patient perspective of continuity of care provides important insight into the delivery of care. The study highlights the importance of person-centred care irrespective of migration background though it may be beneficial to have an awareness of areas that may be of more specific concern to immigrants and refugees. *Conclusions*: The study sheds light on concerns specific to immigrants and refugees in a framework of continuity of care, but also commonalities across the patient groups.

## 1. Introduction

Immigrants and refugees have come to represent a considerable proportion of the population in Denmark; in 2011 they constituted 7.7% of the population [1]. These groups are vulnerable to the development of certain types of mental illness [2,3,4,5,6,7,8,9], are more frequently subjected to coercion [10,11,12,13], and may have limited knowledge of the organisation of the healthcare system, which implies the pathways to treatment become more complicated [14]. Such findings warrant that special attention is given to the care immigrants and refugees receive as they may experience additional vulnerabilities in their contacts with the health care system.

Immigrants and refugees are formally entitled to health care services on the same terms as Danish citizens residing in the country [15]. The majority of services are free of charge to the patient, but cost-free outpatient psychiatric care requires a referral from a general practitioner. Treatment provided by psychologists in private practice requires fee-for-service, but the cost can be subsidised with a referral from a general practitioner when the patient fulfils certain predefined criterions [16].

A large proportion of patients with severe mental illnesses will require long-term treatment, including both inpatient and outpatient care. Continuity of care is frequently highlighted as a way to improve treatment for patients with mental illnesses [17,18]. However, there is no conceptual clarity in the area and many different definitions exist [18] or the concept might not be defined at all [19,20]. Another critique of studies on continuity of care is that it is often assessed by using quantitative measures, such as the proportion of patients referred to community services after discharge [21]. Such measures disregard the patient’s perspective in investigations of continuity of care [22,23,24], even though this perspective can provide important insight into encounters between patients and health professionals. Previous qualitative studies focusing on continuity of care in the psychiatric health care system has mainly been carried out for findings to feed into the development of a questionnaire [25,26] and has not focused on including immigrants and refugees [25,26,27]. The model of continuity of care chosen as a basis for this study consists of four domains, namely, accessibility, individualized care, relationship base and service delivery [22]. Continuity of care is generally considered a central element in high quality care and the concept is particularly important in regard to vulnerable groups such as immigrants and refugees as members of these groups often lack the knowledge and resources to compensate for less than optimal care. Therefore, the aim of this study is to explore continuity of care in the psychiatric health care system from the perspective of the patients, including vulnerable groups such as immigrants and refugees.

## 2. Methods

### 2.1. Terminology

In this article, the term ‘ethnic Danes’ refers to persons born in Denmark by parents who are Danish citizens and also born in the country themselves. “Immigrants” are persons born in a country outside Denmark and who have immigrated to the country themselves in contrast to “descendants” who are persons born in Denmark and whose parents have immigrated to the country. “Refugees” are persons who have been granted official protection in the country.

### 2.2. Eligibility Criteria 

Patients for this study were recruited through a community psychiatric centre in an urban area in the vicinity of Copenhagen and with high proportions of immigrants and refugees in the uptake area. To be eligible to participate in the study patients had to be receiving treatment in the selected community psychiatric centre and be in a stable condition at the time of the interview. Patients had to be ethnic Danes, immigrants, refugees, or descendents—according to the above definitions of the terms—to be eligible to be included in the study. Asylum seekers were excluded from the current study as their access to health care treatment is managed in a separate system.

### 2.3. Recruitment Procedure for Interviews

The patients invited to participate in the study was selected in collaboration with the healthcare professional responsible for treatment to ensure that participation would not be harmful to the patient. The researcher also participated in consultations and assessed the eligibility of patients if this was allowed by the patient. An information leaflet describing the study was developed and translated into the languages of the largest patient groups of foreign origin in the community psychiatric centre, and was available in Danish, English, Turkish, Urdu, and Arabic. The leaflet was developed at a literacy level that the participants could understand. The patients were given the leaflet when they were initially invited to participate in the study. The patient was recruited through a municipal psychiatric centre that offers social activities, but no medical treatment, and had previously been receiving treatment in the community psychiatric centre selected for the study. A diverse group of adult patients with differing migrant backgrounds (immigrants, descendants, refugees and ethnic Danes) were recruited for the study.

### 2.4. The Interviews 

Fifteen patients were included and interviews were conducted with four immigrants, one descendant, five refugees and five ethnic Danish patients. Two pilot interviews with a patient were conducted in June and August 2011, respectively, to assess the applicability of using illness narratives. A total of 19 interviews were conducted with some patients being interviewed twice. The interviews were illness narratives that utilized elements from the semi-structured interview format and methodology in consideration of the vulnerability of the patient group. It was assumed that the narratives of some patients might be very disconnected or that some may have difficulty conveying their experiences. The patients were asked to tell their stories, starting with their first contact with the healthcare system due to mental health problems, and up until the day of the interview. The interview focused on following the patient’s story and at the same time paying attention to what the patient expressed as important in his or her contact with the healthcare system. Towards the end of the interview, the patient and the interviewer outlined the patient’s contacts with the healthcare system in order to clarify the course of events. This was used as a way to further elicit specific areas of interest in encounters with the healthcare system. However, the interviews in this study do not provide an exhaustive description of patients’ contacts with the healthcare services for their mental illness. Instead, they provide insight into the events that stand out clearly for the patients through the entire course of treatment. The interviews lasted between 39 and 107 min. Interpreters were used according to the patients’ preferences and they were able to choose between face-to-face interpretation and phone interpretation.

### 2.5. Fieldwork

Data for this study were collected as part of the first author’s fieldwork in a community psychiatric centre in an area close to Copenhagen that is home to a high percentage of immigrants and refugees. The fieldwork was mainly carried out between November 2011 and March 2012. The author was typically in the community psychiatric centre between two and four days per week and shadowed the health professionals. The main purpose was to facilitate recruitment for subsequent interviews by meeting the patients in person. This approach was chosen due to the vulnerability of the group of patients included in the study. Patients with mental illness may experience obstacles to engage in additional activities and to overcome potential language differences with immigrants and refugees in the recruitment process. The fieldwork also served as a way to learn about the mental health field and determine which patients were able to take part in the interviews. Due to extensive work in building relations and rapport with staff and patients a single community psychiatric centre was selected as a basis of recruitment.

### 2.6. Theoretical Framework

The theoretical framework employed in this article is inspired by a conceptualisation of continuity of care by Joyce and colleagues. This theoretical model was chosen as it was specifically developed based on literature on continuity of care in the psychiatric field. Therefore, it was assessed to be more suitable to the current study than conceptualisations of continuity of care in the general health care system. Overall, four domains included in the conceptualisation of continuity of care are: accessibility, individualised care, relationship base and service delivery [22]. The four domains are made up by several subdomains, which again include lower level categories. As the model involves several levels and is rather complex a simpler representation of the model was selected for analyses in the current study (see Table 2). In this study, the domains in the model serve as the main inspiration of the analysis, but the model is also assessed and evaluated in relation to the patient perspective of continuity of care as part of the results section.

### 2.7. Analysis

The data were analysed using qualitative content analysis as described by Graneheim and Lundman [28]. The interviews were read several times to get a sense of them as a whole. Subsequently, meaning units, *i.e.*, coherent pieces of the text related to the unit of analysis, were selected, condensed and assigned codes and categories in an iterative process. The analytical framework of continuity of care described above was used for further analyses. Thus, the next step of the analysis was to group the categories derived from the data into the four domains of continuity of care in the field of psychiatry: accessibility, individualised care, relationship base and service delivery. This analysis focuses on elements of continuity of care in the psychiatric healthcare system that the patient perspective can provide information on. The analyses included an awareness of commonalities across immigrants, refugees and ethnic Danes, but also of conditions specific to immigrants and refugees.

### 2.8. Theoretical Premises of the Study

Qualitative methods were employed to provide in depth insights and nuances of how and in what way the patient perspective could contribute to an understanding of a selected theoretical framework on continuity of care. The purpose of the qualitative study is not to be able to generalise to the entire population nor does it claim that the analytical categories in this study is of relevance to all patients, but it provides more in depth insight into what is at stake for patients when data is analysed in the framework of continuity of care. However, this does not imply that the experiences of patients in this study would not be of relevance to other patients. On a similar note, we do not intend to make any universal claims on the differences and similarities we observe between immigrants, refugees and ethnic Danes in our data. We do, however, analyse how the analytical categories of relevance to continuity of care unfolds to the patients included in our study. The analytical categories we find to be of specific concern to immigrants and refugees in our study may still be of relevance to some ethnic Danes, but it has not played a large role in the interviews with ethnic Danes in our study.

The patient interviews were mainly narrative in character. Illness narratives focus on the individual’s story and are particularly useful for obtaining illness accounts. It is a gentle method which is well suited to be used with vulnerable groups as the focus of the interview is on what the participant find important [29,30]. Narratives do not aim to provide an exact record of what has happened in a given situation, but represents one possible version of the events, and deals with how people understand past actions and ascribe meaning to them [31]. Therefore, narratives are not meant to be used as a means of verifying or validating what actually took place in a given situation [31] even if the researcher had been present in the situation. However, as the main interest in this study was on the experiences of the patients it is regarded as a suitable method for exploring the course of treatment from the patient perspective. In line with the above premises of qualitative methods and illness narratives we have used a reflexive approach throughout the process of data collection and analysis to ensure the validity of our findings. Thereby, we have sought to critically assess the approach, collection and analysis of data throughout the study in line with the approach to qualitative methods suggested by Stige, Malterud and Midtgarden highlighting the need for ‘reflexive dialogue’ as an important part of the research process [32]. As part of this process the findings were presented to and discussed with other researchers in the fields of psychiatry, medicine, anthropology and public health on several occasions to validate the analytical categories and ensure that these were grounded in the empirical data.

### 2.9. Ethics

Approval for this study was obtained from the Danish Data Protection Agency (approval No. 2010-41-5598). The study was carried out in accordance with the ethical principles of the Declaration of Helsinki [33]. The information leaflet describing the study, which had been translated into the most common languages of the patient population in the community psychiatric centre, ensured that patients could read about the study and take their time to consider whether they wished to participate if they had any reservations upon invitation. It was stressed verbally that participation was voluntary and declining would have no effect on treatment. Written consent was obtained from patients prior to the interview and it was stressed that all statements would be anonymous and that their consent could be withdrawn at any time. Great care was taken by the researcher not to put pressure on patients, and their willingness to participate was assessed continuously. The majority of patients included in the study appeared comfortable sharing their stories and expressed that they were used to it. A few were anxious about participating and extra time was taken to ensure they felt comfortable with the situation. The patients were given a small gift as an appreciation of their participation. They were not informed about this beforehand. Any names appearing in the article have been changed to maintain anonymity.

## 3. Results

The characteristics of the patients are presented in Table 1, and Table 2 provides an overview of the main findings. The results section presents the findings of the study in the domains of the theoretical model. Though the domains are presented separately, we recognise that they are intertwined. Findings of specific concern to immigrants or refugees are pointed out when relevant, but if nothing else is stated, the findings are common across the groups. Themes common to the groups are highlighted in italics in the results section.

**Table 1:** Characteristics of participants in the study.

ID	Age	Sex	Diagnosis (Self-Report)	Source of Income	Migration Background	Country of Birth	Ethnicity (Self-Identified)	Religion	First contact with the Psychiatric Healthcare System	Number of Interviews	Place of Interview
1	46	F	Depression, schizotopy, PTSD	Sickness benefit	-	Denmark	Danish	Atheist	1993	2	Municipal psychiatric centre
2	52	M	Depression	Incapacity benefit	Immigrant	Morocco	Undisclosed	Muslim	2005	2 ^1^	Psychiatric centre
3	47	M	Depression	Social security	Immigrant	Nigeria	Yoruba	Christian	Do not remember	1 ^2 ^	Psychiatric centre
4	45	F	Depression	Social security	Refugee	Iran	Undisclosed	Muslim	Do not remember	1	Psychiatric centre
5	39	M	PTSD	Incapacity benefit	Refugee	Bosnia	Undisclosed	Muslim	Do not remember	1	Psychiatric centre
6	30	M	Undifferentiated schizophrenia, ADHD	Incapacity benefit	-	Denmark	Danish	Christian	App. 2005	1	Psychiatric centre
7	26	M	Paranoid schizophrenia	Incapacity benefit, supplementary work	Refugee	Iraq	Iranian	Muslim	Teenage years	1	Patient’s residence (group home)
8	30	M	Paranoid schizophrenia, brain damage	Social security, supplementary work	Immigrant	Pakistan	Undisclosed	Muslim	App. 2007–2008	1	Patient’s residence (group home)
9	25	M	None	Sickness benefit	-	Denmark	Danish	Atheist	2010	1	Psychiatric centre
10	58	M	Not aware of own diagnosis	Incapacity benefit	-	Denmark	Danish	Atheist	1979	1	Psychiatric centre
11	50	F	Not aware of own diagnosis	Incapacity benefit	Refugee	Iraq	Kurdish	Muslim	Do not remember	2	Psychiatric centre
12	49	M	Schizophrenia	Incapacity benefit	Refugee	Turkey	Kurdish	Undisclosed	1990	2 ^3^	Psychiatric centre
13	26	F	Postnatal depression	Graduate student	Descendent	Denmark	Danish	Muslim	2011	1	Patient’s residence
14	33	F	Paranoid schizo-phrenia	Flex job ^4^	-	Denmark	Danish	Atheist	App. 2000	1	Psychiatric centre	
15	37	F	Depression	Social security	Immigrant	Turkey	Turkish	Muslim	2007	2	Psychiatric centre	

Note: ^1^ Phone interpreter used in second interview; ^2^ Interview carried out in English; ^3^ Phone interpreter used for the first interview and face-to-face interpretation for the second interview; ^4^ A flex job is a type of employement granted by a municipality for persons who have reduced ability to work due to illness.

**Table 2:** Main findings compared to the model of continuity of care by Joyce *et al.* [22].

Model by Joyce *et al.* (2004) [22]		Data from Interviews	
Domains	Subdomains	Categories from Interviews	Conditions Specific Concern to Immigrants and Refugees
Accessibility addresses ‘spatial and temporal conditions that facilitate continuity of care’ [22]	Temporal access Geographic access Barriers to access	Feeling rejected by the system Interrupted course of treatment Transition between services Assistance required to obtain contact with the healthcare system	Lacking awareness on mental illness Obligations towards children may hinder hospitalization
Individualized care ‘addresses the extent to which service providers are sensitive to the personal, social and cultural circumstances of patients in addition to their clinical needs’ [22]	*No sub domains defined in the model.*	Excessive focus on medication by health professionals Exageration of statements and behavior Patints sensemaking of the illness	Trauma Extra vulnerabilities Taboo concerning mental illness
Relationship base ‘addresses the quality of the relationship between patients and service providers’ [22]	Relationship to the system Relationship to the provider	Relationship with the individual health professional Availability of staff	*No differences identified.*
Service delivery ‘addresses the policies, structural features, and procedures that treatment systems and services require to achieve continuity of care’ [22]	System administration Program delivery Provider actions	Flexibility and responsiveness in the system Transfer of information between units Detachment between patient and treatment initiatives Coordination with social services	Coordination with social services extends to immigration papers

### 3.1. Accessibility

Based on the interview data with patients, four main categories described their experiences in accessing treatment: feeling rejected by the system, interrupted course of treatment, transition between services, and assistance from others required to obtain contact with the healthcare system.

Several patients described situations in which they *felt rejected by the system*, e.g., if their concerns were not taken seriously by health professionals. It can be difficult for patients to describe their experiences and, particularly in initial contact, their symptoms and feeling of unease may be described in very subtle terms. Though the situation might not appear very serious to the health professional, that is not necessarily the case for the patient. A young ethnic Danish man described how, after a period of feeling depressed and having isolated himself, he contacted his general practitioner. However, he was not able to get a referral nor was he offered any kind of follow up by the general practitioner. Describing the situation he said:

At this time, I started thinking, well maybe it is just me who thinks something is wrong. And then I break down completely (…)

*(ID 6, male ethnic Dane)*

After the visit, the patient started questioning whether he was really in need of help, but still he was unable to continue his work and simply stopped showing up. He had planned to take his own life when he ran out of money, but was finally hospitalised when a concerned colleague showed up at his home. Other patients also described how initial rejection by the system or long waiting periods for treatment simply postponed contact to a later time when they were in a far more severe state and needed to be hospitalised – often in a closed ward and admitted through the emergency room. Feeling rejected when trying to reach out for help was described by the patients as humiliating and not being taken seriously.

*Interrupted course of treatment* due to lack of funding also affected access to services. Most often this was seen with treatment by psychologists. Access to psychologists was described by patients as very difficult to obtain, whether this was sought while being admitted or through referral from a general practitioner. Several patients paid for such treatments out of their own pocket, but had to stop as they could not afford the payments. Likewise, some have been granted access through the municipality, but this may also result in frequent interruptions in the course of treatment, as the expiry of funds did not necessarily follow the needs of the patient.

*Transition between services* formed important events for continued access to appropriate treatment. Some participants described transitions between services as unproblematic. However, transitions between different units was clearly shown to be a potentially problematic time for continued delivery of care. Problems with transitions became visible when there was a lack of care or follow up and when transitions failed, severe consequences may follow. A refugee from Turkey described his experiences:

I didn’t know (…) when I got well the doctors told me I could leave. I was discharged. Even though they were all experts no one told me I could get sick again.

*(ID 12, male refugee)*

The patient has been admitted approximately 10 times over the course of several years, but without realizing that he needed further treatment once discharged from the hospital. At the time of the interview, he received treatment on a regular basis from the community psychiatric centre. An ethnic Danish patient also described similar experiences with lack of proper follow up for patients. She was hospitalised after a suicide attempt and was expecting to receive counselling by the hospital. However, she was simply given a couple of brochures with places she could contact for help; no one noticed that the patient did not feel she was able to make use of these options. Though she was already familiar with the suggested facilities, she did not feel comfortable going there, as she used to work close by. In addition, she expressed that it is difficult to initiate contact with new treatment facilities when you do not feel well.

Many also described how they had been in *need of assistance from others*, either realizing that they needed help or gaining access to the healthcare system. Such assistance may come from family, friends or colleagues. In several cases, access to treatment was only granted when the patients returned to the treatment facility, assisted by someone else. Several immigrant and refugee patients expressed that they were not familiar with mental illness before they were diagnosed or admitted involuntarily – sometimes through the forensic psychiatric system – which complicated access to treatment. Two of the immigrant and refugee women also described that lack of social relations can hinder access to treatment. They described how hospitalisation was not an option, as they do not have anyone who was able to look after their children. One of these women did not trust leaving her child with her family and for the other woman the event took place shortly after she arrived in Denmark implying she did not have a network in the country.

In the theoretical framework, the domain of accessibility focuses mainly on timely care, geographical features and barriers to access [22]. In this study, distance to services was not salient in the patients’ accounts of what mattered in terms of being able to access treatment, whereas receiving care in time of need and barriers to access were important. Patients described their experiences of being turned away from the healthcare system if they were not assessed to be ill enough when reaching out for help. Approaching the healthcare system represents a vulnerable position for patients and being turned away without any planned follow up could have serious consequences. To be taken seriously, patients were often dependent on other people to negotiate contact with the healthcare system on their behalf. Specific concerns to immigrants and refugees in this domain included a lack of knowledge of mental illness, which may postpone initiation of treatment, and obligations towards children, which may hinder hospitalisation due to limited social networks in the country.

### 3.2. Individualised Care

The categories that pertain to the domain of individualised care, based on the interviews, include excessive focus on medication by health professionals, exaggeration of statements and behaviour, and the patients’ sensemaking of the illness.

Several patients perceived that some health professionals had an *excessive focus on medication* and that other aspects of their lives were disregarded in clinical encounters. Medication could give rise to tension between patients and health professionals and several patients described difficulties when declining medication. The lack of acknowledgement of the patients’ perspective implied that patients could feel detached from their treatment, and in several cases this led to patients reducing or discontinuing medication without involving health professionals. Accounts of negative encounters with health professionals were often tied to descriptions on a narrow focus on medication and lacking understanding of the patient’s perspective. A few patients felt that their *statements and behaviour were exagerated* to fit with medical symptoms and diagnoses, which made it difficult for patients to come to terms with the diagnostic conclusions of the health professionals.

When *patients make sense of their illness* this was often associated with various life events. For immigrants and refugees, such events may include cultural issues and traumatic events as illustrated in the quotes below.

That is why I became sick. I didn’t get sick all of a sudden. My childhood, youth. A different country. Language. My mother’s illness. It is very difficult to be the daughter in a Muslim family.

*(ID 15, interview 1, female immigrant)*

(…) then there was the war in Bosnia. I have seen many bad things and I have been to war myself and then I came to this work and he [the boss] has bullied and harassed me and then everything fell apart.

*(ID 5, male refugee)*

The fact that patients’ made sense of their illness and encounters with the health care system in light of their broader life experiences was very salient in patient descriptions. However, several patients expressed that they felt that their view of the illness has been disregarded by health professionals. Though this was a common experience across the patient groups, there are examples specific to immigrant and refugee patients. Past experiences may influence patients’ encounters with the healthcare system, providing relevant information for the interpretation of specific patient reactions. A female refugee, who had previously been incarcerated in Iraq, expressed her distress about being in the hospital.

And the door was shut. It was a closed ward. It comes to my mind again, how we were in prison in my country. The door was shut. It was very difficult.

*(ID 11, female refugee)*

One of the female immigrants spoke of similar distress regarding the prospect of hospitalisation. Though the episode had taken place many years ago, even explaining the situation caused a very strong emotional reaction during the interview. The patient was a victim of severe violence throughout her childhood. The violence violence, carried out by different family members, had continued into her adult life. As a child, she was subjected to punishments such as being locked up in a dark basement by her mother and later she was locked up by her physically abusive ex-husband in their house for two years. These descriptions of how hospitalisation evokes feelings of former imprisonment with some of the immigrant and refugee women emphasize the importance of individualised care. In these cases, the patients’ prior experiences highly impacted what kind of treatment they were able to receive in the healthcare system.

Though all patients had experienced vulnerability and strong social adversity throughout their lives, additional vulnerabilities were very apparent in the narratives of immigrants and refugees. Immigrant and refugee patients who experienced war traumas were severely marked by these experiences, and may also have been the victims of extreme violence, loss of family members, and might still be worried about family members left behind in countries involved in conflicts. Some were marked to such a degree it was still very difficult for them to talk about their experiences.

The immigrant and refugee patients in our study reported that mental illnesses were considered a taboo and were not accepted in their culture. However, they expressed that their close family members were aware of their mental illnesses and had been mainly supportive. However, extended family and friends were typically not aware that they were suffering from a mental illness as they feared the gossip of more distant relations in the community. Also, they explicitly referred to patient confidentiality as why they could confide in health professionals.

In the model, individualised care addresses to what extent health professionals are sensitive to circumstances that go beyond the clinical needs of the patients and in particular focus on personal, social and cultural conditions [22]. The interview data with patients supported the conclusion that conditions in addition to clinical needs are important in the treatment of patients. However, the patients perceived that their care was often focused solely on adjustments to medication, whereas their own understanding of their illness was highly connected with life events and social circumstances. Previous experiences may also have had an impact on encounters with the healthcare system. Conditions specific to immigrants and refugees in the domain of individualised care included the existence of trauma, extra vulnerabilities, and mental illnesses were taboo with extended family members and acquaintances.

### 3.3. Relationship Base

In the interview data, the categories in the domain relationship base included the *relationship with the individual health professional* and *availability of staff*. The most salient finding in the whole study was the importance of the *relationship with the individual health professional*, implying that satisfaction with treatment was very person dependent. Pivotal encounters with a specific health professional may have taken place in initial contact with the healthcare system, but patients may also have been receiving treatment through years before they encounter a health professional with whom they developed a deeper connection. A woman from Iraq who has had contact with many different providers in the healthcare system over many years and who has attempted suicide several times described her relationship with the healthcare professional currently responsible for her treatment:

I want somebody who understands us. As I said, from the day I [have] spoken to Sarah I am much better. Really good. When I start having problems, I say to myself: go to Sarah (…). I love her. But I would like to say I got help only from this place, psychiatric centre. And I am happy to [go], I don’t want to stop.

*(ID 11, female refugee)*

Positive descriptions of encounters with health professionals were often tied to a feeling of being understood as a person and having one’s concerns taken seriously by the health professionals. Some even referred to specific health professionals as family members to express the close connection. A young woman of Turkish descent explained how being well received and met with an understanding of her situation influenced her decision to accept treatment from the community psychiatric centre:

(…) most people I met were really good at making me feel calm [saying] ‘it is quite normal how you feel and what you did’ so I opened up to meeting new people and having the session (…). Had I been told something else (…) or I had felt that they didn’t understand me I would have said no to starting at the community psychiatric centre.

*(ID 13, female descendent)*

In contrast, patients also described how a lack of understanding from health professionals had a negative impact on their encounter. These examples show the importance of connections with individual providers, but also help to illustrate how vulnerable alliances with the patients may be. 

*Availability of staff* and time to talk with patients were also raised as important in relationships with healthcare professionals, and the lack of these elements could give rise to patient frustration. Patients reported several accounts of health professionals, particularly psychiatrists, being out of reach for them. This gave rise to feelings of neglect and not being worthy of spending time with them, which led to frustration in patients. The patients expressed that availability of staff and being able to communicate with them within a foreseeable amount of time gave a sense of security and a feeling of being prioritised.

In the model, the domain of relationship base focuses on the quality of the relationship between patients and health professionals. The relationship may be with the system as a whole as well as the individual health professional [22]. The interview data supported that relationship base is an important element of continuity of care from the patient perspective. The above examples supported the notion that pivotal encounters in the treatment of patients were highly dependent on the individual health professional encountered by the patient. The patients’ accounts illustrate that the degree to which healthcare professionals are receptive to patients may be of great importance regarding the extent to which patients were able to benefit from the services offered in the healthcare system. Availability of staff within a foreseeable time frame was important for patients to feel recognised and safe. Within this domain, there were no differences across the groups.

### 3.4. Service Delivery 

The categories in the patient data included flexibility and responsiveness in the system, transfer of information between units, detachment between the patient and treatment initiatives, and coordination with social services.

*Flexibility and responsiveness* in the system was stressed as important to the patients. Several patients described how being able to adapt the frequency of consultations to their needs was highly valued. In addition, being able to stay in touch with a health professional, while waiting to access treatment with a different provider, was also described as very valuable by patients. Patients also described how responsiveness in the system may be important. One of the patients described how she missed an appointment when she was starting to relapse and the outreach work by the health professionals due to the missed appointment led to her being hospitalised. Other types of outreach work described as positive by patients included visiting hospitalised patients before they initiated treatment in the community psychiatric centre.

*Transfer of information between units* in the healthcare system is a crucial part of service delivery, but often it is not very evident to the patient whether this has happened or not. Some patients did not necessarily see it as a problem that they had to explain their situation to a new health professional, whereas others found it very emotionally straining to explain their situation every time they saw a new provider. Transfer of information between different units and providers may also have unintended negative consequences. One patient felt that the health professionals kept viewing her current problems in light of a diagnosis she was given 20 years earlier. However, it may also become a problem if transfer of information is assumed. In the quote below, the patient had switched to a new general practitioner and did not say anything about his mental illness for a long time, as he assumed the new general practitioner was already familiar with his situation.

I only told him the story after a long time. Okay and how can that be (…)? Well, he didn’t ask me anything. For my part, I thought he knew.

*(ID 2, interview 2, male immigrant)*

Another important issue in service delivery is the *detachment between the patient and treatment initiatives* that sometimes seems to happen. The diagnosis may not necessarily be considered very relevant to the patient, but rather something that pertains to the healthcare system and the healthcare professionals. Likewise, some patients also described that the purpose of treatment was not very clear to them. Sometimes the efforts in the healthcare system may not even be considered “real treatment” and there was a clash of understanding of treatment between the patients and the system:

I don’t know… she sends me to all sorts of strange examinations that really doesn’t have anything to do with my head that blood pressure, cholesterol and all that stuff they come up with, I don’t really see the point of it.

*(ID 10, ethnic Danish male)*

Patients also described how their treatment in the healthcare system extended to *coordination with social services*. This may be in relation to obtaining housing such as a group home, assistance with obtaining financial aid such as social security or incapacity benefits. In the case of immigrants and refugees, these services also extended to assistance with immigration papers for the Danish Immigration Service.

The domain of service delivery is very system focused in the theoretical framework and deals with policies, structural features, and procedures necessary to obtain continuity of care in the healthcare system. The subdomains of service delivery include system administration, programme delivery and provider action [22]. From the patient’s perspective, these elements were not very salient, but they may still be important for the experience of the patient. Flexibility in the system included adapting the number of consultations according to the needs of the patient and a responsive system where health professionals actively seek contact with patients whom have not shown up for their appointment, may also be of importance in the delivery of care to patients. Transfer of information mainly became important when it did not occur. If patients do not comprehend why certain treatment initiatives were carried out, this may lead to a detachment between the system and the patients who were receiving treatment. For immigrants and refugees, coordination of social services also extended to assistance with immigration papers.

## 4. Discussion

Investigating the patient perspective of continuity of care—based on patients with diverse migration experiences and ethnic Danes—enabled us to identify issues of similar experiences in encounters with the healthcare system as well as concerns more specific to immigrants and refugees. We found issues of particular relevance to immigrants and refugees in the domains of accessibility, individualised care and service delivery, but not in the domain of relationship base. All domains included issues shared across the groups. The discussion focuses on some of the most prominent findings in the study of continuity of care from the patient perspective. These findings are discussed by the introduction of new theoretical perspectives which contributes to the understanding of why these are important from the patient perspective.

One of the most salient findings in our study was found in the relationship base domain. The patients placed an immense importance on the *relation to the individual health professional* for the establishment of a meaningful connection between patient and provider. Previous studies have also found the relation to the provider a crucial part of continuity of care [18,26,27]. The inclusion of immigrants and refugees in our study imply that the therapeutic alliance will sometimes be formed across different ethnic and cultural backgrounds. Culture has been found to impact the therapeutic alliance [34] though the effect of corresponding initiatives such as the matching of patient and provider based on ethnicity remains inconsistent [35,36,37,38]. However, scholars have found that positive encounters between patient and providers can be obtained across different cultures and ethnicities [37,39,40]. The patients in our study emphasized that feeling respected and understood by the professional is crucial to establishing a trusting relationship, which is a finding that was salient across the patient groups. This indicates that these elements form an important component of the therapeutic alliance in all patient-provider encounters.

Another very salient finding was from the domain individualised care. In this domain it was very evident that patients incorporate life events when *making sense of their illness*. However, patients in our study describe that they encounter solutions in the healthcare system that highly value medication, whereas they often experience a lack of recognition of their own perspective on their illnesses. Still, the course of mental illnesses is increasingly recognized to be a complex interplay of biological, psychological and social factors [5,34,41,42,43,44,45] and chronic illness has been described as such a disruptive life event that it can influence the entire biography of a patient [46,47]. This offer support to a stronger focus on the patients’ lived experiences of illness in the health care system [30]. Kleinman distinguishes between the concepts illness and disease. Illness is the patient’s lived experience of symptoms and disability and disease is the physician’s representation of the disorder after having reworked the patient’s account into a medical framework [30]. He places much importance on the perspective of the patients and suggests that the patient’s knowledge is considered an alternative to the biomedical perspective. Thereby, the patients’ knowledge should not be disregarded as irrelevant or incomplete in encounters with physicians [30]. It has been found that the inclusion of context and how patients create meaning in their illnesses can influence patient care and augment the validity of the diagnosing of mental illness [30,48,49]. Scholars have shown that inclusion of cultural and other contextual elements from patients’ lives lead to different diagnoses and treatment plans for immigrant patients [49]. Showing an interest in the patient’s perspective can be crucial for creating trust between a patient and a provider [49] even if the view is not necessarily shared by the physician. Our data strongly support the importance of paying attention to the patient perspective and including contextual information in the diagnosing and treatment of mental illnesses for immigrants and refugees as well as ethnic Danish patients.

In the domain individualised care also saw a salient theme which was more profound to immigrants and refugees. *Additional vulnerability* due to exposure to prolonged and repeated traumatic events by some refugees and immigrants rendered patients extremely sensitive and highly influenced by their experiences, which also became apparent in their contact with the psychiatric system. Though the existence of previous trauma in refugees is thoroughly documented [45], it is often missed in a clinical setting [50]. Our findings show that previous trauma may also be evident for immigrant patients, which again supports a focus on the context of the individual patients.

Another important theme specific to immigrants and refugees was found in the domain accessibility. The findings from our study show that access was influenced by a *lack of knowledge of mental illness* for some of the immigrant and refugee patients, implying that access to psychiatric treatment was—in some cases—initiated through the forensic psychiatric system. Though the ethnic Danish patients were not necessarily able to recognise that their symptoms were related to mental illnesses, they had an understanding of mental illness as a concept. Previous literature has found immigrants and refugees along with ethnic minorities to have more complicated access pathways to psychiatric treatment [51,52,53,54], and immigrants and refugees underutilise mental health services despite great need [55,56]. This may be due to stigma, lack of understanding of mental illness, and different explanatory models [34,56,57]. Access to care is multifaceted and may be influenced by a number of factors, but our data support the fact that immigrants and refugees may experience additional obstacles in access to psychiatric treatment.

### 4.1. Discussion of Methods

An advantage of the current study is that it gives a strong voice to the patient perspective by using interviews with patients as the main data collection. However, this choice for the collection of data also meant that some patients from the community psychiatric centre were excluded from participation as taking part in an interview also sets certain demands for patients e.g., many were not well enough to carry out an interview. Another advantage of the study was that the main author was able to spend an extended period of time in the field, which provided valuble information for contextualizing findings based on interviews and served to ensure the validity of the analysis.

The number of interviews carried out varied between patients. However, this is not expected to have influenced data substantially as the researcher sought to obtain good rapport with the patient and create a comfortable setting and atmosphere. Some patients had problems concentrating for too long due to their illness and the medication and were invited for a follow-up interview if the researcher experienced they had more to contribute, but were not able to complete the first session.

The use of interpreters entails a risk of miscommunication, ommissions or wrong translations. However, we decided the most important for our study was that patients were not excluded due to lacking proficiency in Danish. As the patients interviewed spoke different languages it was not possible to perform any extensive training with the interpreters, but we sought to remedy the use of interpreters by having initial conversations where the approach of research interviews as well as the topic was introduced to the interpreters. The patients would choose whether they preferred face-to-face or telephone interpretation. The advantage of face-to-face interpretation is that all people present can have a sense of facial expressions and other non-verbal body language in assessing the situation and the translation. For the researcher it is easier to have an understanding of whether what is being translated corresponds to what the patient has said. On the other hand, the telephone interpretation might imply that it is easier for the patient to speak more freely on sensitive topics if the interpreter cannot see them.

Negative accounts from patients with mental illness may easily be disregarded with references to their distorted sense of reality or lack of disease-insight. However, mental illness is episodic, implying there will be fluctuations in the severity of the disease [58]. During the interviews, the patients in our study were also able to talk about how they remembered psychotic episodes and distance themselves from the view of the world they had during such episodes. Scholars have also conducted research on patients with mental illness experiences in even more severe circumstances e.g., such as patients’ experiences of being subjected to coercion [59,60,61].

## 5. Conclusions

In this study, we explored continuity of psychiatric care, investigating the concept from the perspective of ethnic Danes, immigrants and refugee patients. The analysis of continuity of care from the patient perspective is based on the theoretical framework consisting of the domains accessibility, individualized care, relationship base and service delivery. The domains included in the framework are all found to be important from a patient perspective. However, some discrepancies were found between the subdomains in the model and the issues valued from a patient perspective. Based on the model, the study sheds light on issues of specific concern to immigrants and refugees, but also on commonalities across the patient groups. Having health professionals acknowledge conditions specific to the individual patient and feeling respected and understood by health professionals was found to be important across patient groups. The study shows that the course of treatment for patients with mental illnesses includes such complex relations that a focus on the patient perspective is necessary to deliver high quality care. Furthermore, we found that having experienced trauma that give rise to psychiatric consequences was not solely restricted to refugees as we also saw examples among some immigrants. Though traumatic experiences may be more widespread in the refugee population it is important for health professionals to keep in mind that immigrant patients may also have been subjected to similar experiences.
